# Effects of analgesia on the response to a noxious stimulus in Norway lobsters (*Nephrops norvegicus*)

**DOI:** 10.1038/s41598-026-41687-w

**Published:** 2026-04-13

**Authors:** Eleftherios Kasiouras, Guiomar Rotllant, Albin Gräns, Per Hjelmstedt, Lynne U. Sneddon

**Affiliations:** 1 Department of Biological and Environmental Sciences, University of Gothenburg, PO Box: 463, Gothenburg, 405 30 Sweden; 2 Institut de Ciéncies del Mar (CSIC), Passeig Marítim de la Barceloneta 37, Barcelona, 08003 Spain; 3 Department of Applied Animal Science and Welfare, Swedish University of Agricultural Sciences, PO Box 463, Gothenburg, 405 31 Sweden

**Keywords:** Decapod crustaceans, noxious stimuli, analgesia, animal welfare, behaviour, stress, Neuroscience, Physiology, Zoology

## Abstract

There is evidence that decapod crustaceans have the capacity to experience aversive states associated with nociception, highlighting the need for humane treatment. Behavioural and physiological responses to nociceptive stimuli may be reduced with analgesic drugs, yet little is known for decapods about effective options. In this study, the impact of electric shock on behaviour and physiology was investigated in Norway lobsters. Behaviour was monitored from before the shock to two hours after, with and without drugs with analgesic properties. Haemolymph and nervous tissues were analysed for stress indicators, including lactate, glucose, and gene expression. Electric shock induced tail flipping responses, absent in sham groups, however this was significantly reduced in animals treated with a local anaesthetic and an analgesic; suggesting these responses were mediated by nociception. Aspirin and lidocaine were effective at reducing this shock-related behaviour, however aspirin injection increased grooming immediately after administration, with elevated haemolymph lactate and downregulated gene expression in ganglionic tissues. Overall, the acute shock stimulus did not produce long lasting effects, but both drugs reduced tail flipping during shock. These results underscore the potential of these drugs for laboratory use during invasive procedures, but also the broader ethical responsibility to incorporate welfare strategies in crustacean handling and stunning protocols.

**Supplementary Information:**

The online version contains supplementary material available at 10.1038/s41598-026-41687-w.

## Introduction

Animal welfare has rapidly evolved in the 21^st^ century due to the advancements of biology and neuroscience^1^. Most scientists acknowledge that all vertebrates are able to experience pain^2^. Today, the attention has largely shifted towards groups of invertebrates, such as the decapod crustaceans, fuelling changes in the welfare considerations of these animals^3–6^. Notably, certain Australian territories, Austria, New Zealand, Norway and Switzerland have banned boiling of live lobsters, and the UK has already recognized decapod crustaceans as sentient beings^7^ although see opinions in Diggles et al.^8^ that have been addressed by other authors with respect to pain in fishes [e.g.^9–11^]. Despite that, confirming nociceptive processing in animals remains a challenge^2,12^: the main issue is the inherent inability to communicate with animals. Hence, the scientific approach to answering the question includes a comprehensive analysis of behaviour, physiology, as well as molecular and biochemical indicators associated with nociception-induced responses since these indicators are not seen during innocuous stimulation^2,13^.

Pain and nociception are distinct but related phenomena. Pain can be defined as “an unpleasant sensory and emotional experience associated with, or resembling that associated with, actual or potential tissue damage”^14^. Nociception is defined as “The neural process of encoding noxious stimuli”^15^ where injury causing stimuli are transduced into action potentials and conveyed to the central nervous system. Transduction is typically rapid and may be followed by an instantaneous reflex withdrawal response^2^. Arguably, based on these two definitions, nociception can occur without pain; however, healthy animals cannot experience sensory pain (i.e., tissue damage) without nociception. Thus, nociception underpins the negative affective component of pain^16^. Examples of noxious stimuli include electric shock, injection of chemicals that excite nociceptors, high and low temperatures and high mechanical pressure^17^. Pain can potentially occur when specific receptors, termed nociceptors, detect actual tissue damage and the information is conveyed to the central nervous system where it is processed, which can often result in a negative psychological experience termed pain^18^. Therefore, when assessing whether an animal experiences nociception and/or pain, it is important to consider behavioural, neurobiological and physiological changes during or after a noxious event and whether drugs with analgesic properties prevent these responses^2^.

Low voltage electric shock has been widely used in research in learning paradigms as a punishment, because it is a noxious stimulus that can evoke avoidance and defensive responses^19–21^. Thus, it would be important to understand how electric shock may affect the behaviour and physiology of decapod crustaceans. So far, the effects of electric shock in decapods have been studied behaviourally where crabs given an electric shock in a specific shelter exhibited avoidance behaviour towards that shelter subsequently^22^. Other behaviours can be utilised to indicate stress in decapods, such as total activity of the animals. For example, tail flipping is a particularly important indicator of stress because it is a rapid escape response to imminent danger^23^. Behavioural responses are the primary way that animals react to stress and to avoid potential injury and/or danger some decapod crustaceans can perform a series of rapid tail flexions or tail flips to move away from dangerous or injury causing stimuli^24^. Additionally, the effects of electric shock in crayfish were also studied both behaviourally and physiologically where the levels of serotonin and dopamine were measured^25,26^. Exposure to electric shock also increased activity in crabs^27^. However, more data are needed to comprehensively assess how electric shock, using different electrical parameters, influence the behaviour and physiology of decapod crustaceans.

Physiological measurements, including stress indicators and brain gene expression, are imperative because nociception is inherently stressful and alters the physiology of the central nervous system (CNS) activity^28,29^. During stress glucose concentrations rise due to elevated blood catecholamines^30^ and lactate can increase as a consequence of anaerobic respiratory activity since metabolic demand cannot be met solely from aerobic metabolism, which occurs during the stress response^31^. The CNS of decapod crustaceans consists of a ladder-shaped series of ganglia that are connected to each other by nerves presenting several fusions along the chain from the eyestalk to the telson^32^. Key stress-related genes can be measured after stressful events to assess neurobiological responses. Neurotransmitters like excitatory acetylcholine and inhibitory GABA (gamma-aminobutyric acid) regulate nervous system function in these ganglia^33,34^. The CNS controls endocrine, immune, and stress responses, affecting genes like crustacean hyperglycaemic hormone (CHH) and somatostatin, which regulates cardiac and foregut activity during stress^35,36^. Stressful and/or noxious stimuli may alter the expression of these genes in specific ganglia in decapod crustaceans, necessitating targeted investigations of these tissues.

To investigate whether electric shock may cause nociceptive responses to decapods, similarly to other taxa, it is vital to test whether the administration of drugs with analgesic properties can prevent any nociceptive responses. This approach, a key criterion for assessing nociception and/or pain in animals, has not been thoroughly explored in decapods crustaceans^2,37–39^. A few studies have evaluated the effects of lidocaine in decapods, which is a local anaesthetic with analgesic properties that blocks the sodium channels of sensory neurons including nociceptors providing a numbing sensation^40–42^. Lidocaine can be administered by immersion in the water and proved to be an effective analgesic in *Macrobrachium americanum*^43^. Lidocaine has been validated as providing pain relief to other animal groups^44^. Other commonly used analgesic drugs are the non-steroidal anti-inflammatory drugs (NSAIDs) such as aspirin (acetylsalicylic acid) which are often investigated in pain research^45^. Aspirin inhibits the activity of the enzyme cyclooxygenase (COX) which normally leads to the formation of prostaglandins (PGs) that cause inflammation^46^. COX enzymes have been identified in decapod crustaceans^47^ and relevant pathways involving prostaglandins have been characterised^48^ so there is the potentiality of aspirin to exhibit similar mechanisms of actions noticed in other taxa^49^, but inflammation has not been studied in decapods. Although to the best of available knowledge, this study represents the first instance of aspirin being used to modulate nociception in decapod crustaceans. Hence, the use of these drugs can strengthen the argument that decapod crustaceans may exhibit reduced nociceptive responses to potentially harmful stimuli^50,51^.

Therefore, the aim of the study was to investigate two criteria for assessing nociceptive responses in animals in the commercially important decapod species the Norway lobster (*Nephrops norvegicus*). The following hypotheses were established to assess the impact of electric shock: Electric shocks will induce responses in decapod crustaceans that are consistent with nociception (Hypothesis 1); and administration of drugs with analgesic properties, such as lidocaine or aspirin, will mitigate the escape responses induced by electric shock in decapod crustaceans, suggesting their potential in modulating nociceptive responses (Hypothesis 2). We expect acute responses to electric shock as it is only noxious for the duration of the shock and studies have shown few long-term effects on nociceptive behaviour, except avoidance behaviour in training paradigms^19,52^.

## Materials and methods

### Animals

Norway lobsters, *Nephrops norvegicus* (mean (± SE) weight = 106.04 ± 1.66 g; carapace length = 51.36 ± 0.30 cm; *n* = 105) were obtained from a local anonymous commercial supplier from an area north of Gothenburg, Sweden. Only males were obtained in the beginning of August 2022. The Norway lobsters were transported by car to the experimental aquarium at the University of Gothenburg, 90 min after pickup. They were placed into cool boxes with cool packs and wet tissue paper to isolate the animals from one another. All animals survived and they were held for a period of three weeks in three stock tanks (one tank of 200 × 200 × 40 cm and two tanks of 142 × 142 × 40 cm; 40 animals in large tank, 32 and 33 in the two smaller tanks) to acclimate to laboratory conditions before they were used in experiments. During this time the Norway lobsters were fed three times per week *ad libitum* using cooked mussels obtained frozen from a local supermarket, chopped into small pieces. Saltwater was made by mixing distilled water with marine salts (Instant Ocean salt, Sarrebourg, France) to 34 ± 1 ppt. Water parameters were maintained within the following values: temperature 10 ± 1 °C, pH 8.1 ± 0.1, NH_4_^+^ < 0.1 mg/L, NO_2_^-^ < 0.1 mg/L, NO_3_^-^ < 20 mg/L and continuous aeration was provided via air stones. Half of the tank was covered from above to provide a darker area and PVC pipes were available for each animal to use as a “burrow” to provide shelter. Lobsters were checked visually daily to ensure good health and welfare. The light/dark cycle was 12:12 h light: dark with a dim up and down of 30 min to reflect dusk and dawn.

Animals were chosen haphazardly and placed into a 25 L bucket of saltwater from their system (stock tanks) and transferred to an individual tank in an adjacent room with the same water supply. Then lobsters were immediately transferred to their individual tanks (60 × 22 × 45 cm) where all lobsters showed recovery, by feeding and behaving normally. Beforehand the individual tanks were filled with water from the stock tank, to maintain similar water quality. The tanks were supplied with saltwater made by mixing distilled water with marine salts (Instant Ocean salt, Sarrebourg, France) to a salinity of 34 ± 1 ppt. Additionally, the water was filtered in each tank using sponge filters and aeration was provided through an air stone connected to a compressed air supply. The water was changed daily up to 1/3 of total volume and replaced with new saltwater to maintain good water quality. Following the transfer, the Norway lobsters were allowed to acclimate for 7 days and were fed three times per week *ad libitum* with mussels. Uneaten food, if any, and waste were removed manually each day by using a siphon, approximately 45 to 75 min after feeding. The first experiments took place in September but due to the need to increase sample size another experimental round took place again in September and early October. All experiments were conducted at 09:00 a.m. and all animals were euthanized around 01:00 p.m. All lobsters were in the intermoult stage as defined by Aiken^53^. Moreover, the condition of the animals was visually assessed with the vigour index, according to Albalat et al.^54^. All animals looked healthy and were classified as category A.

### Treatment groups

**Fig. 1:**
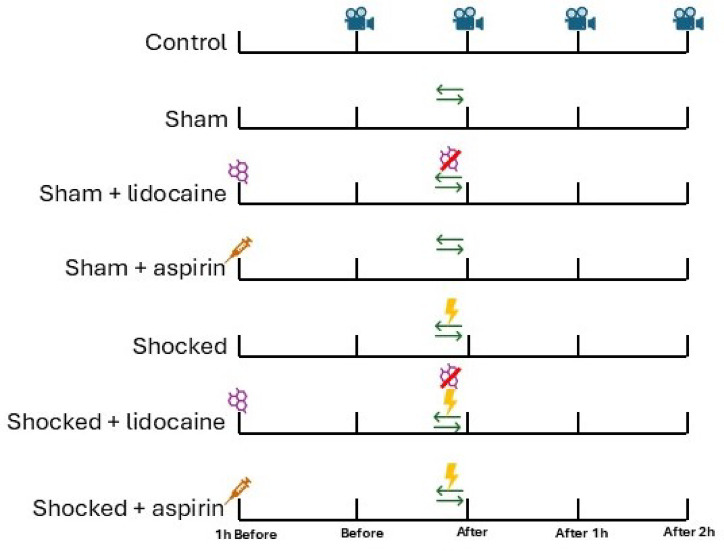
Schematic of the experimental procedure for each treatment group. Animals were filmed in the home tanks for 15 min. Animals were transferred to shock tank from their home tanks and back. Animals received an electric shock in the shock tank (9.09 V/m).  Lidocaine was dissolved in the home tank water (80 mg/L). Lidocaine was removed from the home tanks.  Aspirin was injected into the animals (10 mg/kg).

After 7 days of acclimation to the individual tanks, animals were assigned to a treatment group in a haphazard manner (*n* = 15 per group). The groups were Control (undisturbed), Sham (handled but no shock), Sham + lidocaine, Sham + aspirin, Shocked, Shocked + lidocaine, Shocked + aspirin. All the treatments that each group received are summarised in Fig. 1.

Animals were filmed in the home tanks for 15 min. Animals were transferred to shock tank from their home tanks and back. Animals received an electric shock in the shock tank (9.09 V/m). Lidocaine was dissolved in the home tank water (80 mg/L)  and was removed during the transfer to the shock tank. Aspirin was injected into the animals prior to the shock (10 mg/kg).

The experiment started by administering analgesics to the animals, one hour prior to the initiation of the study, before the first behavioural observation was made. Lidocaine (80 mg/L) (Sigma-Aldrich, Saint Louis, USA, Lidocaine hydrochloride monohydrate) was administered via immersion by dissolving the drug in the water of the home tank. To prevent changes in water pH, sodium bicarbonate (Sigma-Aldrich, Saint Louis, USA, Sodium hydrogen carbonate) was added as a buffer (80 mg/L). A pilot study, not included in this manuscript, showed that sedation occurred at doses of 120 mg/L and above, but not at 80 mg/L. Animals that were administered with aspirin (Sigma-Aldrich, Saint Louis, USA, Acetylsalicylic acid, 99.0%) received 10 mg/kg via injection on the arthrodial membrane at the coxa of the fourth pereiopod with a sterile needle (25 G) and syringe (1 mL), one hour prior to the experiment. Aspirin was dissolved first in ethanol (Solveco, Roseberg, Sweden, Ethanol, 99.5%) 40 g/100mL in room temperature and then diluted with sterile 0.9% NaCl (Sigma-Aldrich, Saint Louis, USA, Sodium chloride) till final concentration 1 mg/0.1 mL for the injection. The shocked groups were exposed to an in-water electric shock for 10 s using a 50 Hz AC source, delivering an electric field of 9.09 V/m and a current density of 0.44 A/dm^2^ at 10 °C and a water conductivity of 48,000 uS/cm. More specifically, the electrical setup was a custom-built device assembled by Ace Aquatec (Ace Aquatec Ltd. Dundee, United Kingdom). The system consisted of a variable AC transformer connected to an isolating transformer, capable of delivering a 50 Hz sinusoidal AC wave form at a selected root-mean-square (RMS) voltage. To control the duration of the exposure, a timer was connected to the variable AC transformer’s power supply. Additionally, the electrical setup was connected to two stainless steel plate electrodes (470 × 1 × 140 mm) and the distance between these electrodes was 115 mm. The electrodes were placed inside the experimental tank (480 × 122 × 188 mm, 11 L), in salt water (34 ppt, conductivity of 48000 uS/cm). Finally, the voltage and current were monitored with an oscilloscope (Fluke 123B Industrial Scope meter, Fluke Europe B.V., Eindhoven, Netherlands) and a current clamp (Fluke, model 80i-110s), during the electrical exposures, to ensure a consistent electric field.

### Behavioural recordings

On the day of experimentation each animal was filmed using digital cameras (Sony, HDR- 167 CX240E, Handycam, 9.2 megapixels, Tokyo, Japan), for 15 min before (baseline), immediately after return to the home tank, and after 1 and 2 h post shock or handling. For the drug treated groups the pre-treatment recording occurred one hour after administration to allow recovery from the disturbance. The three sides of the home tanks were covered with black opaque plastic to avoid visual disturbance from adjacent tanks. Immediately after the recording, each animal was transferred individually using a 25 L bucket of saltwater to an adjacent room where the electric shock took place. This included the Sham groups, which were not shocked but received the same handling. The water in the shock tank was changed after each trial. The water in the individual tanks was replaced during this time in all tanks but specifically to remove the lidocaine dosed water for Sham + lidocaine and Shocked + lidocaine. Immediately after the noxious stimulus, the animals were transferred back to their individual tanks and were recorded for 15 min. Recordings were also made 1 and 2 h after the noxious stimulus to assess recovery. Different behaviours were recorded to calculate the overall activity of the animals. The behaviours were, walking forward, reverse walking and crossing, turning, rearing up, climbing/claw display^55^. All these behaviours were summed as total activity time (s). Additionally, grooming/scratching was observed at a high rate in the drug treatments so total time of grooming was also calculated (s). During the electric shock the animals were also filmed with a camera placed above the shock tank, and the number of tail flips (n) was measured, when electric shock was applied. There were two independent observers analysing the videos to obtain behavioural data, so a Pearson correlation and Intraclass correlation were used to confirm the robustness and reliability of the data (see Supplementary Information (S.I.)).

### Physiology and molecular biology

**Fig. 2:**
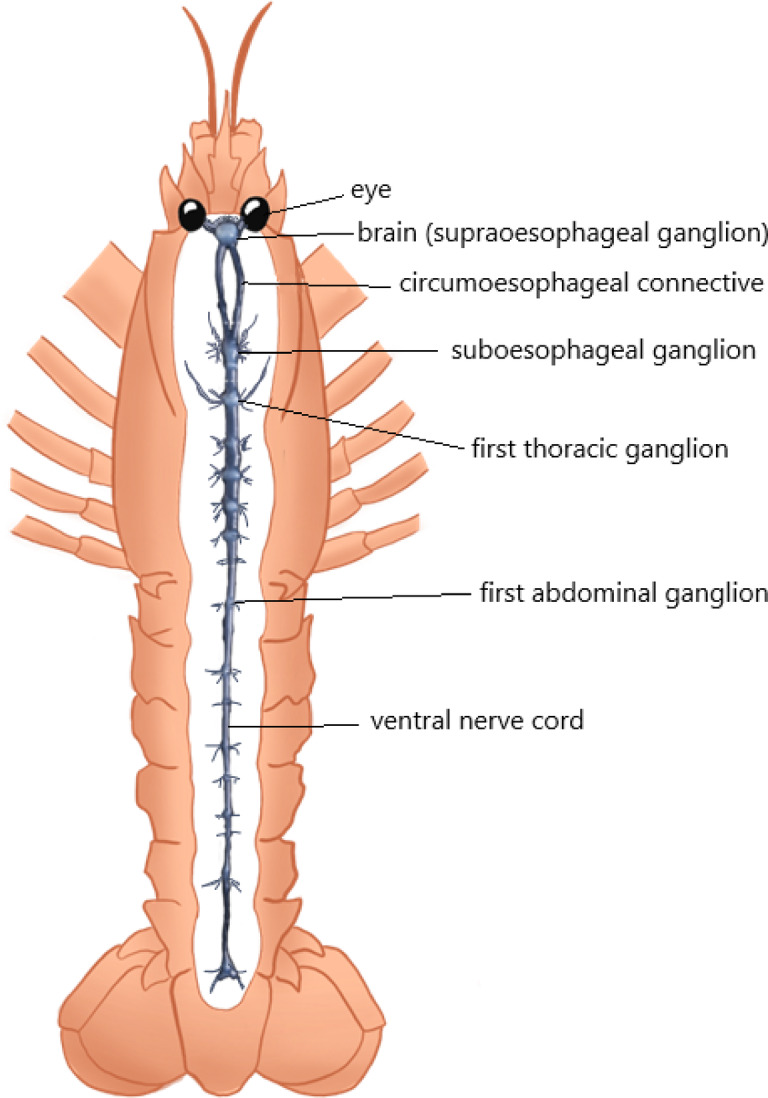
The characterization and segmentation of the 4 main areas of ganglia of *Nephrops norvegicus*, that were used to assess the gene expression (created by Lisa Carlsson and reproduced with permission).

Following the behavioural experiments haemolymph was extracted with a sterile needle (25G) and syringe (1 mL) from the arthrodial membrane at the coxa of the on the fourth pereiopod to assay for glucose and lactate, indicators of the secondary stress response^56^. Commercially available enzymatic kits were used (Glucose (HK) Assay Kit, GAHK20, product n°G3293, Sigma-Aldrich, St Louis, USA, and Lactate Assay Kit, In-struchemie, product n°2864, Delfzijl, The Netherlands, respectively). Then their carapace length (to 0.1 cm) and wet weight (to 0.1 g) were measured. Afterwards the animals were sampled for nervous system tissues for qPCR analysis. From the anterior to the posterior part of the body we find in sequence, firstly the X-organ sinus gland in the eyestalks followed by the supraoesophageal ganglia (SOG). Then the circumoesophageal connective links the SOG (dorsal) to the suboesophageal ganglia (ventral). After that the nerve cord continues to the thoracic ganglia and the abdominal ganglia that reach till the end of the gut, close to the anus (Fig. 2).

To ensure humane and efficient nervous tissue sampling, Norwegian lobsters were placed on ice to induce anaesthesia or sedation, reducing pain and stress while preserving tissue integrity, as supported by previous research^57^. Subsequently, the central nervous system was extracted including the (brain) supraoesophageal ganglia (SOG) with the circumoesophageal connective, the suboesophageal ganglia (subOG), thoracic ganglia (TG) and the abdominal ganglia (AG) (Fig. 2). CHH and neurotransmitter receptors acetylcholine, GABA B1, GABA B2, and somatostatin were measured in specific ganglionic areas, since these relate to stress and were identified in the transcriptome produced by Rotllant et al. (2017)^58^, using blastp similarity-based search, and then were functionally annotated with InterProScan. Elongation factor 1 α (elf1α) and glyceraldehyde 3-phosphate (GADP) were used as reference genes^58^. Primers were designed with OligoAnalyzer program. The primer’s list and characteristics can be seen in Table 1. From the central nervous system RNA was extracted using the commercial kit (QIAGEN, Germantown, MD, USA, RNeasy Lipid Tissue, Mini Kit). Then cDNA was synthesised and qPCR was performed following similar methodology of Lind et al.^59^, quantifying the expression of the five genes: CHH, receptor for acetylcholine, GABA B1(alpha), GABA B2(beta) and somatostatin. Initial quality checks demonstrated a low yield of RNA; therefore, three samples were pooled together from three different animals so there was enough quantity of RNA extract for fabricating cDNA and the qPCR. The total number of RNA samples were 5 per group.

**Table 1:** Primer information on the genes that were used to test gene expression in the four main ganglionic areas across the seven different treatment groups of Norway lobster (*n* = 15).

Housekeeping genes	Forward (5’-3’)	Reverse (5’-3’)	Size (bp)	T_m_ (^o^C)
Elongation factor 1a	CAA CAA GAT GGA CAG CAC AGA	CAG AAA TGG GGA GGA TGG	315 bp	58
GADP	AGT CCC CTC GCA ACA CCT	CCA TCC TCC ATC TTC ACC TC	337 bp	60

Target genes	Forward (5’-3’)	Reverse (5’-3’)	Size (bp)	T_m_ (^o^C)
Acetylcholine receptor	GAC GAG AAG AAC CAG ATC C	GTA AGT CCA GGA GCC GAA C	324 bp	60
CHH	TAA GGG CGT GTA TGA CCG	ACA TTG ACG GAA CAC TCG G	300 bp	60
GABA B1 receptor	AGT TCT CGG GTA CAA CAG GA	GGC GTT AGT GGA GGA CAT	250 bp	60
GABA B2 receptor	AGG CAA CTA CTC TCG GCT	GGC GTT AGT GGA GGA CAT	205 bp	60
Somatostatin receptor	CTC CAA GAT GCA GAC GGT CA	GTG ACA GAC GGC GAT GTA C	225 bp	58

### Statistical analysis

Six video files were found to be corrupt and could not be viewed by a second observer. Thus, these animals were excluded from the data analysis resulting in an n of 13 for each group for behaviour. All of the data for duration of activity and grooming were normally distributed.

To evaluate whether the administration of the drugs with analgesic properties (aspirin via injection or lidocaine in bath) reduces acute aversive escape behaviour (tail flips) in the stunning tank during electric shocks, the number of tail flip responses was compared among three groups: Shocked only, Shocked + lidocaine, and Shocked + aspirin. A Kruskal-Walli’s test was performed, followed by a Dunn’s test.

To investigate whether handling, electric shock, and/or the drugs affected the behaviours in their home tanks (total activity and grooming) across four time points (before, immediately after, after 1 h and after 2 h), four separate two-way repeated ANOVAs with Bonferroni Correction (Bonferroni Post Hoc Test) were conducted. The behaviours served as dependent variables, while the four time points were treated as repeated independent variables. The analysis included three main comparisons: (1) To evaluate the impact of the handling routine and to assess the effect of electric shock, the Shocked group was compared with the Sham and the Control group. (2) To determine whether the drugs, or the injection itself, influenced the behaviour of non-shocked lobsters, the Sham groups (Sham, Sham + lidocaine, and Sham + aspirin) were compared. (3) To examine the effects of drugs on the behaviour of shocked lobsters, all shocked groups (Shocked, Shocked + lidocaine, and Shocked + aspirin) were compared.

To compare the effects of electric shock on the haemolymph levels of lactate and glucose, a Kruskal-Walli’s test and Dunn’s test was compared similarly as the behaviour, following the same comparisons. The relative gene expression of the acetylcholine, CHH, GABA B1(alpha), GABA B2(beta) and somatostatin receptors were compared with a Kruskal-Walli’s test and Dunn’s test. Each gene was analysed separately for each ganglionic area. Lactate, glucose, tail-flips, and pooled gene expression were analysed using Kruskal–Wallis tests, as each dataset consisted of a single independent value per experimental unit. Activity and grooming behaviours were measured repeatedly in the same animals and were therefore analysed using repeated-measures ANOVA. Each individual was treated as a subject, and the repeated measurements over time (before, immediately, after 1 h and after 2 h) were treated as the within-subject factor. This approach inherently accounts for the correlation between repeated measurements within each subject. Therefore, additional random effects were not included. Statistics were performed on SPSS (version 29.0, IBM Corp., Armonk, NY, USA). Statistical significance was determined at a threshold of *p* < 0.05.

## Results

### The effects of drugs on the escape behaviour during electric shock

**Fig. 3:**
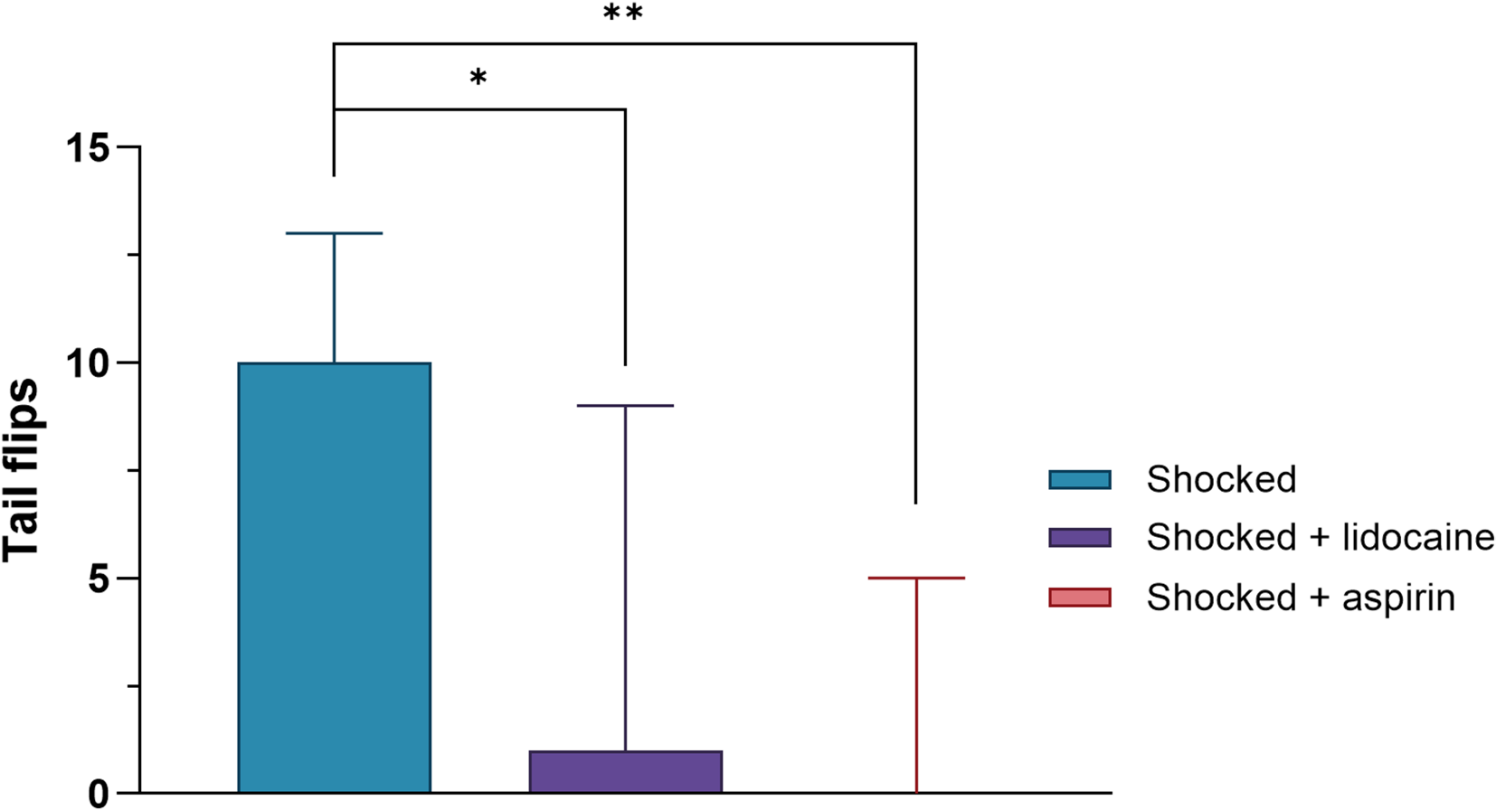
The number of tail flips performed by Norway lobster during the shock across the three electrically shocked groups (Shocked, Shocked + lidocaine, Shocked + aspirin). Median of tail flips (± IQR) (*p < 0.05, **p < 0.01, *n* = 13, per group).

Tail flips were observed exclusively in the shocked animals. Significant differences were found between groups in the number of tail flips performed during the electric shock (df = 2, H = 12.607, *p* = 0.002). Both analgesics, aspirin and lidocaine, reduced tail flips in comparison to the Shocked group (Shocked compared with Shocked + lidocaine, *p* = 0.026; Shocked compared with Shocked + aspirin, *p* = 0.002; Fig. 3). Additionally, the animals that did not receive a shock did not exhibit any tail flipping. In contrast all animals in the shocked group without analgesia (Shocked) exhibited tail flips. Tail flips were observed in only 7 out of 13 animals in the Shocked + lidocaine group and 3 out of 13 in the Shocked + aspirin group. Only 3 animals in Shocked + lidocaine group and 1 in the Shocked + aspirin group exhibited a frequency of tail flips comparable to those observed in the Shocked group.

### Factors affecting the total activity of the Norway lobsters

#### Impact of handling and electric shock on the activity of Norway lobsters

**Fig. 4:**
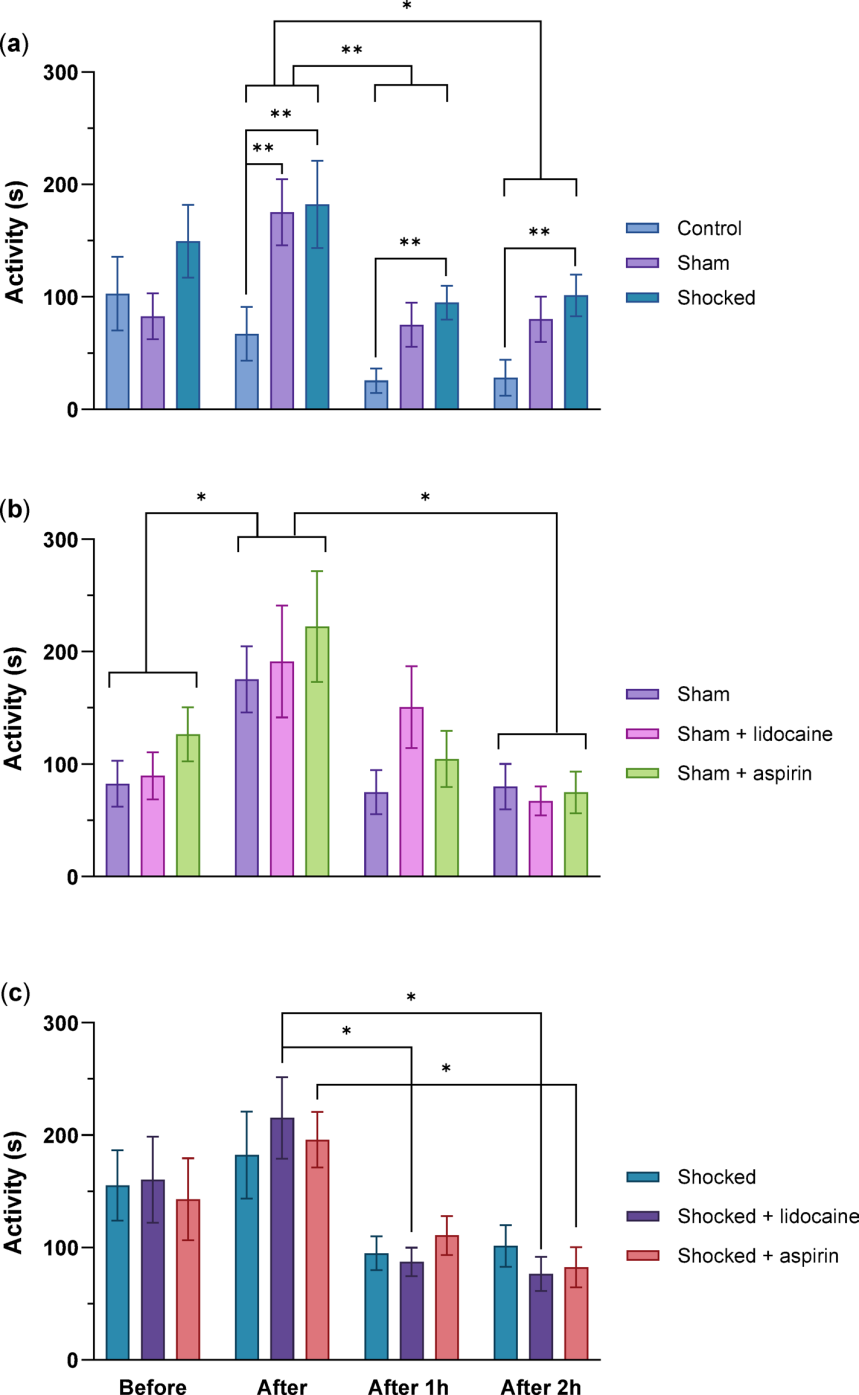
Total activity in Norway lobsters. The effects of; (**a**) the handling routine and the electric shock, (**b**) lidocaine and aspirin administration in non-shocked lobsters and (**c**) lidocaine and aspirin administration in electrically shocked lobsters. Mean total activity (± SE) (*p < 0.05, **p < 0.01, *n* = 13, per group).

The three groups Control, Shocked and Sham were compared to assess the effects of handling and electric shock. There was an effect of time (F_(3,114)_ = 5.834, *p* = 0.008) and there was an effect of the treatment (F_(2,72)_ = 6.482, *p* = 0.008), but there was no interaction between time and treatment (F_(6,228)_ = 1.517, *p* = 0.224). Shocked and Sham animals exhibited higher activity than the Control groups after handling and electric shock but there were no differences between Sham and Shocked animals (after: Control-Sham, *p* = 0.005, Control-Shocked, *p* = 0.006, after 1 h: Control-Shocked, *p* = 0.009, Control-Shocked, after 2 h: *p* = 0.005). In addition, there were differences between time points where Shocked group and Sham groups showing higher activity exactly after the treatment than after 1 h and after 2 h (after-after 1 h: *p* = 0.004, after-after 2 h: *p* = 0.034) (Fig. 4a).

#### The effects of the drugs on non-shocked lobsters

No significant effects of the drugs were observed in non-shocked lobsters, as total activity did not differ between the three Sham groups (F_(2,72)_ = 0.584, *p* = 0.537). There was an effect of time (F_(3,114)_ = 10.170, *p* = 0.001), but no interaction between the treatment and time (F_(6,228)_ = 0.776, *p* = 0.537). Total activity across all three Sham groups was higher immediately after the handling compared to before (*p* = 0.027) and after 2 h (*p* = 0.004; Fig. 4b).

#### The effects of the drugs on shocked lobsters

No significant effects of the drugs were observed in the shocked lobsters, as total activity did not differ among the three shocked groups (F_(2,38)_ = 0.005, *p* = 0.976). There was a significant effect of time (F_(2,38)_ = 12.439, *p* < 0.001), but no interaction between the treatment and time (F_(4,152)_ = 0.348, *p* = 0.777). In the Shocked + lidocaine group, animals exhibited significantly higher total activity immediately after the electric shock compared with 1 h and 2 h after (*p* = 0.031 and *p* = 0.022, respectively). Similarly, the Shocked + aspirin group exhibited significantly higher total activity immediately after the electric shock compared to after 2 h (*p* = 0.019). Both groups had similar total activity before the treatment (*p* = 0.989; Fig. 4c).

### Factors affecting grooming in the Norway lobsters

#### Impact of handling and electric shock on the grooming of Norway lobsters

**Fig. 5:**
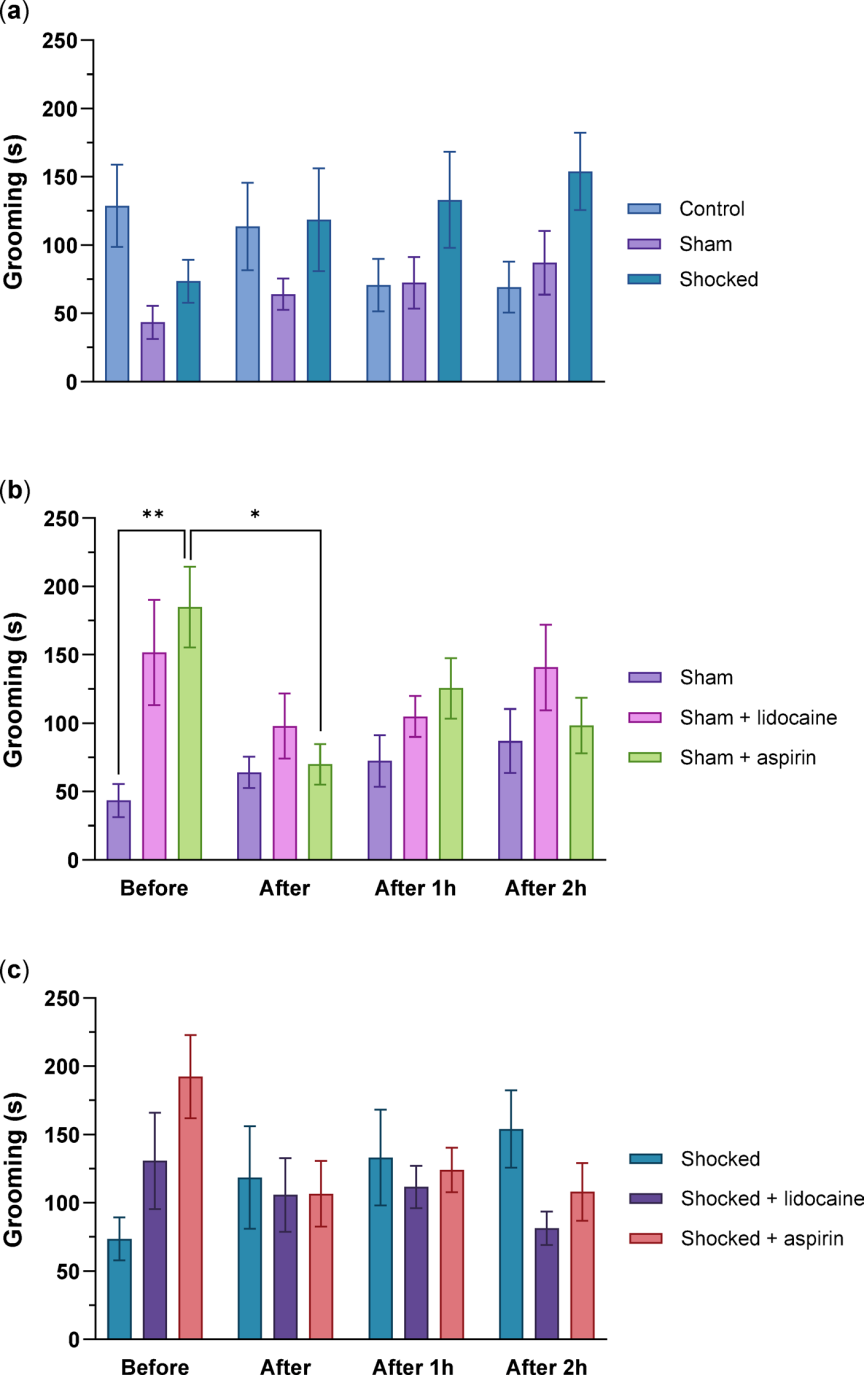
Grooming patterns in Norway lobsters. The effects of; (**a**) the handling routine and the electric shock, (**b**) lidocaine and aspirin administration in non-shocked lobsters and (**c**) lidocaine and aspirin administration in electrically shocked lobsters. Mean total grooming (± SE) (*p < 0.05, **p < 0.01, *n* = 13, per group).

When the three groups Control, Sham and Shocked were compared there were no differences in grooming between these three treatments (F_(3,114)_ = 2.929, *p* = 0.073). Additionally, there were no effects of time (F_(2,72)_ = 0.735, *p* = 0.500) and there was no interaction between time and groups (F_(6,228)_ = 2.230, *p* = 0.087) (Fig. 5a).

#### The effects of the drugs on non-shocked lobsters

Grooming differed significantly among the Sham groups (F_(2,72)_ = 4.966, *p* = 0.026) and across time points (F_(3,114)_ = 3.180, *p* = 0.043), but there was no significant interaction between time and treatment group (F_(6,228)_ = 2.452, *p* = 0.080). The Sham + aspirin animals exhibited higher grooming activity than the Sham group before the handling (*p* = 0.007). Within the Sham + Aspirin group, grooming was more frequent during the baseline measurement compared to immediately after handling (*p* = 0.014) (Fig. 5b).

#### The effects of the drugs on shocked lobsters

No significant effects in grooming patterns were observed among the three electrically shocked groups (F_(2,38)_ = 0.639, *p* = 0.508), across time points (F_(4,152)_ = 0.446, *p* = 0.669), or in the interaction between time and treatment group (F_(5,36)_ = 2.274, *p* = 0.084) (Fig. 5c).

### Lactate and Glucose concentrations in the haemolymph of Norway lobsters

#### Lactate

#### Impact of handling and electric shock on the lactate levels of Norway lobsters

**Fig. 6:**
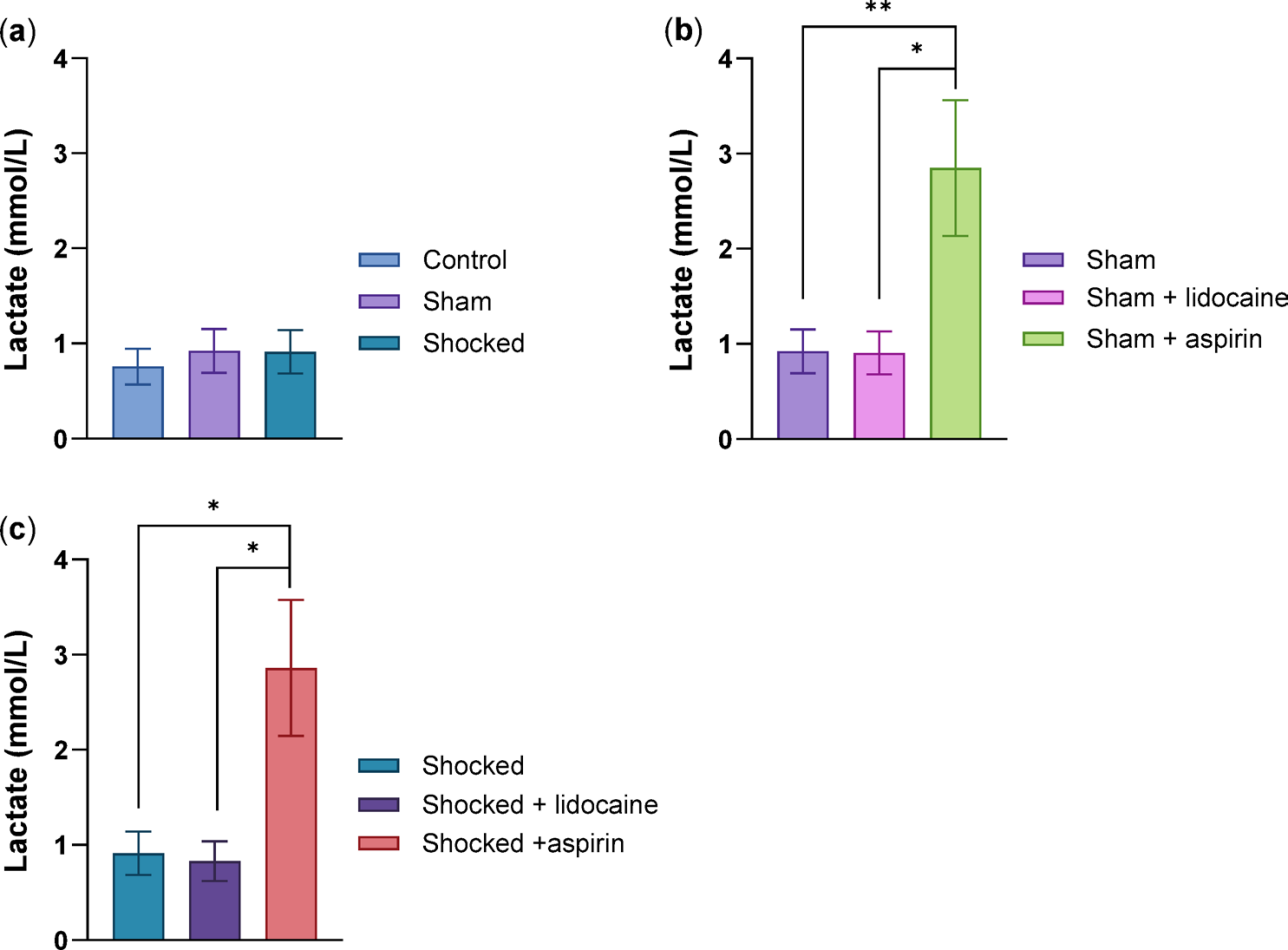
Haemolymph lactate concentrations in Norway lobsters. The effects of; (**a**) the handling routine and the electric shock, (**b**) lidocaine and aspirin administration in non-shocked lobsters and (**c**) lidocaine and aspirin administration in electrically shocked lobsters. Median total lactate levels (± IQR) (*p < 0.05, **p < 0.01, *n* = 13, per group).

When the three groups Control, Sham and Shocked were compared, there were no differences in lactate levels between these three treatments (df = 2, H = 0.228, *p* = 0.892) (Fig. 6a).

#### The effects of the drugs on non-shocked lobsters

Lactate levels differed significantly among the Sham groups (df = 2, H = 11.661, *p* = 0.003). The Sham + aspirin animals had higher levels of lactate in comparison to Sham and Sham + lidocaine groups (Sham + aspirin-Sham, *p* = 0.007, Sham + aspirin-Sham + lidocaine, *p* = 0.012) (Fig. 6b).

#### The effects of the drugs on electrically shocked lobsters

Lactate levels also differed between the shocked groups (df = 2, H = 10.034, *p* = 0.007), with the Shocked + aspirin group having higher levels of lactate than Shocked and Shocked + lidocaine group (Shocked + aspirin-Shocked, *p* = 0.013, Shocked + aspirin-Shocked + lidocaine, *p* = 0.027) (Fig. 6c).

#### Glucose

Glucose levels did not differ significantly in any of the comparisons above (S.I. Fig. S3).

### Differential gene expression in the CNS of the Norway lobsters

#### Brain and suboesophageal ganglia

No significant differences were detected in the brain ganglia between the five different genes across the seven groups. In the suboesophageal ganglia the expression of GABA B1 and GABA B2 differed among the groups but no further differences were found when the groups were compared with Dunn’s test (see Supplementary Fig. S4a and Fig. S4b).

#### Thoracic ganglia

**Fig. 7:**
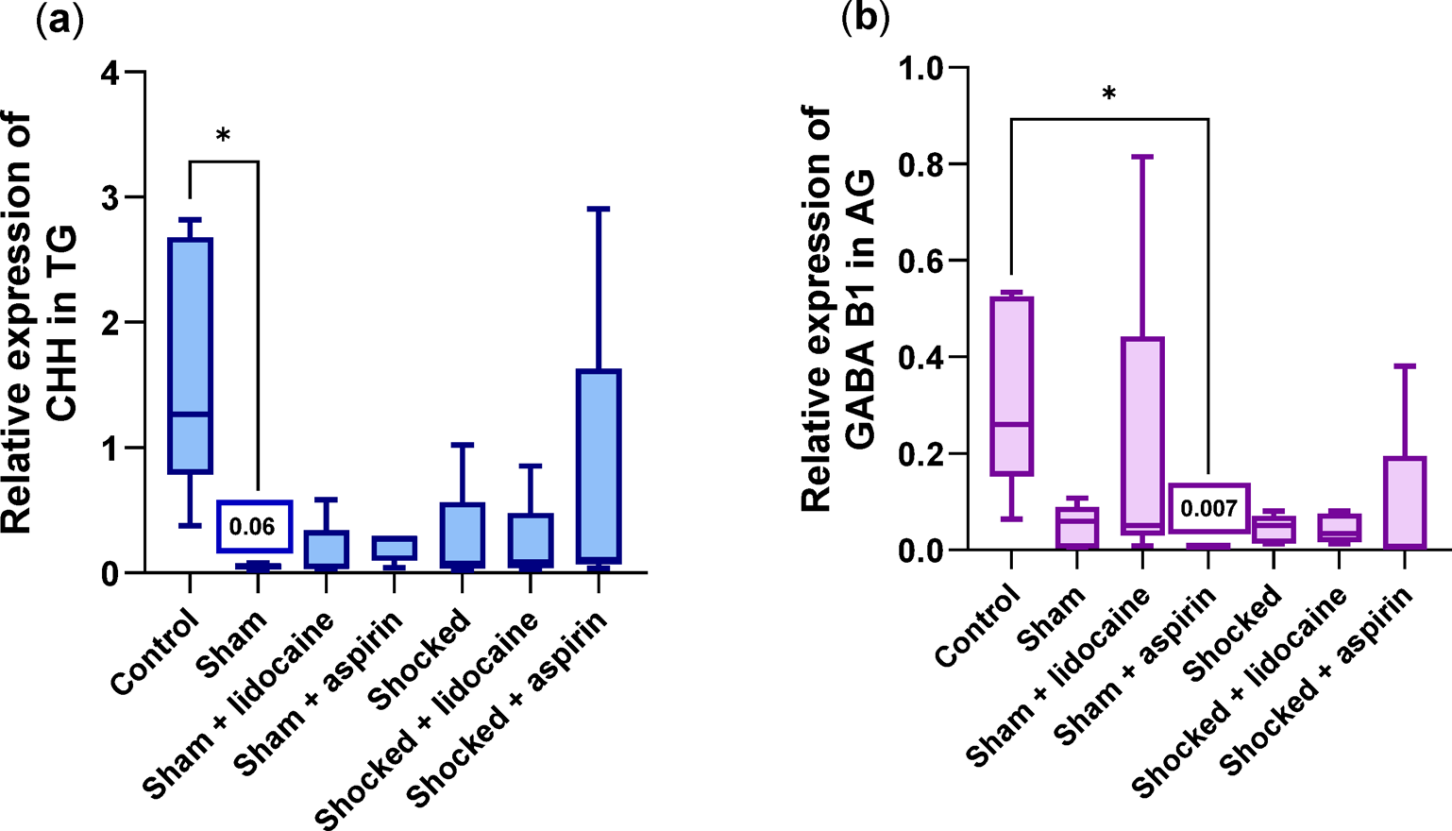
Box plots showing the differential gene expression of (**a**) CHH in thoracic ganglia and (**b**) GABA B1 receptor in abdominal ganglia, of Norway lobster across the seven groups. Box plot (within the box, the horizontal lines denote the median values; boxes extend from the 25th to 75th percentiles of each group’s values; the vertical extended lines represent the 95% range of values) (*p < 0.05, *n* = 5, per group).

In the thoracic ganglia, CHH was the only gene with a significant treatment effect (CHH: df = 6, H = 14.153, *p* = 0.028). CHH was up regulated in the control group compared to the sham group (sham – control, *p* = 0.021) (Fig. 7a). Expressions levels of the other genes showed no significant difference among groups (acetylcholine: df = 6, H = 10.741, *p* = 0.097; GABA B1: df = 6, H = 8.569, *p* = 0.199; GABA B2: df = 6, H = 4.620, *p* = 0.593; somatostatin: df = 6, H = 8.855, *p* = 0.182).

#### Abdominal ganglia

In the abdominal ganglia, only GABA B1 showed significant differential expression among groups (acetylcholine: df = 6, H = 9.448, *p* = 0.150; CHH: df = 6, H = 9.799, *p* = 0.133; GABA B1: df = 6, H = 14.489, *p* = 0.025; GABA B2: df = 6, H = 6.365, *p* = 0.384; somatostatin: df = 6, H = 7.247, *p* = 0.299). GABA B1 expression was up regulated in the control compared with the sham + aspirin group where the expression was downregulated, indicating that the injection of aspirin affected the expression of receptors in the abdominal ganglia (control – sham + aspirin, *p* = 0.018; Fig. 7b).

## Discussion

The study investigated the effect of electric shock as an acute noxious stimulus and the role of analgesic drugs on both the behaviour and physiology of the Norway lobster. Under normal conditions (Control), animals exhibited low levels of activity and grooming. However, during electric shock, tail flipping was observed indicating a nociceptive response. Aspirin and lidocaine significantly reduced this behaviour, likely by reducing inflammation, if it occurs, and modulating nociceptive neuronal activity respectively, consequently reducing the perceived noxious stimulus. Handling alone also induced stress evident by increased activity and grooming. These findings suggest that the electric shock used here constituted a negative experience for the Norway lobster, but the impact was acute and further changes on the behaviour was due to a combination of the stress of handling and/or the electric shock. Although both drugs seem to mitigate the acute effect of electric shock, the injection of aspirin also affected behaviour and stress physiology suggesting there are side-effects measured as increased grooming before the application of the shock, elevated lactate and downregulation of neural gene expression specifically GABA B1.

### Hypothesis 1

Our results support Hypothesis 1 since electric shock did affect the Norway lobster. These results are consistent with the concept of nociception in animals, since the electric shocks induced clear behavioural responses that were not seen in non-shocked animals. Shocked lobsters exhibited increased tail flipping during the electric shock, aligning with previous findings in crustaceans exposed to aversive stimuli^27^. Similar behaviours were also observed in the freshwater prawn *M. americanum* during a potentially painful procedure, eyestalk ablation^43^. Tail flips, grooming/rubbing, other escape behaviours and limping have been suggested as indicative of distress and nociceptive responses in decapods and other animals^2,60,61^. For instance, when formalin was applied to shore crab claws, the animals shook their claws and performed various movements including increased grooming of the affected claw^62^. In the present study activity patterns were altered by both sham and shock treatment suggesting handling, transfer to the shock equipment and return to the home tank was stressful. The increased activity patterns post-shock in sham and shocked groups, are comparable to another study when shore crabs that were exposed to brief electric shock exhibited higher activity patterns in comparison to control animals^27^. Similar observations have been also noticed in other taxa such as mice and fish where electric shock impacted both their physiology and behaviour^63,64^. However, although acute effects of electrical shock were recorded long term effects were not evident. Electric shock is an acute stimulus and although it is used in learning paradigms^19^, its potential to induce long-lasting negative effects, beyond learned avoidance, may vary depending on factors such as the strength of the stimulus. Future studies should explore noxious stimuli that exert their effects over a longer period to compare with electric shock. Increased grooming of an injured site can indicate stress and irritation. For example, cuttlefish injected with acetic acid, performed grooming at the site of injection which was reduced by lidocaine^65^. Grooming patterns were correspondingly affected, where animals exhibited higher grooming even after two hours from the shock, but sham animals also performed elevated grooming suggesting this is a stress response to handling and other studies have found increase grooming because of stress^60,66^. Prolonged responses including grooming were also reported when acetic acid was applied to the eyes of shore crabs^67^. The high activity and grooming patterns in sham and shocked animals decreased over time, so animals exhibited a rapid recovery. Even though, a short-term effect of the electric shock was noticed, in our study, the application of electrical shock, a well-established noxious stimulus^68^, elicited rapid tail flipping, with approximately 10 tail flips occurring within just 10 s. Furthermore, the administration of analgesics significantly reduced the number of tail flips from, about 10 to almost zero. This pharmacological modulation supports the interpretation that the tail flip responses to electrical shock are mediated by nociceptive pathways rather than mere mechanical stimulation or light disturbances^69–71^. If this was just a simple effect of stimulation of motor neurons by the electric shock, then aspirin and lidocaine would have no effect. Together, these findings indicate that electric shock induced tail flips that are reduced by drugs with analgesic properties. This evidence fulfils one of the criteria that is necessary for the definition of animal pain^2^ and provides information relevant to understanding the capacity for nociception in decapod crustaceans.

Electric shock did not affect haemolymph glucose concentrations in the Norway lobster. Only lactate levels were higher in both groups injected with aspirin (Sham + aspirin and Shocked + aspirin), indicating that aspirin or the injection, might induce a stress response, but we do not see this in the Shocked group. However, haemolymph was taken over 2 h after the handling and shock treatments when behavioural responses were close to pre-treatment suggesting the animals had recovered. Further, if the shock was exerting its effect through muscle contraction alone, we would see a high value of lactate in shocked animals, which suggest that electric shock was indeed exciting nociceptors. Future studies should use electrophysiological approaches to confirm this. Alternatively, previous research has shown that lactate increases when CHH increases, and that electric shock can cause that increase without a corresponding increase in activity^27^. Moreover, it would be interesting to analyse samples from the hepatopancreas to assess stress responses as an additional biomarker, because it is a key organ involved in metabolism, detoxification, and energy storage^72,73^. Since nociception is processed in the CNS, analysing gene expression is as equally important as is the analysis of secondary stress indicators. Similarly, the expression of the investigated candidate genes was also unaffected except the expression of GABA receptor B1, a key regulator of inhibitory neurotransmission, which was also reduced in aspirin-treated sham animals, possibly reflecting disrupted neuronal activity or enhanced excitation^74^. These findings suggest that stressors, including drug administration, can alter gene expression in neural regions and thus the handling procedure in this study did induce stress. While our results provide evidence of altered gene expression, earlier sampling would be critical to capture acute transcriptional changes immediately following stress exposure. Electric field may be important in mediating the tail flip response. In the study of Roth and Øines (2010)^75^, a two-way stun was used in edible crabs, where the electric field was 530 V/m initially (1 s) and then 170 V/m (2 min). In the current study only 9.09 V/m was used. So, future studies may also wish to explore the impact of increasing the electric field or investigate a relatively longer lasting stimulus to determine the impact on behaviour, stress physiology and CNS gene expression. This information may be relevant to understanding the use of electric stunning as a humane method when stunning or killing crustaceans^4,56,75^.

### Hypothesis 2

Lidocaine and aspirin reduced the tail flip responses performed by the Norway lobster during the electric shock. Thus, these drugs appear to be effective in alleviating nociceptive responses induced by electric shock. Lidocaine effectively reduced tail flipping without significant behavioural or physiological side effects, except with a non-significant increase of grooming when the drug was provided, indicating a small irritation, which subsided within an hour, making it a promising candidate for managing nociceptive responses in decapods. Aspirin injection, while reducing tail flips, elicited elevated grooming and increased lactate concentration in the haemolymph, suggesting it may have stress-related side effects and an unintended behavioural impact. Interestingly, Sham groups injected with aspirin also displayed increased grooming prior to shock, potentially due to irritation or a stress response but this remains to be tested. This increase in lactate may be attributed to the injection itself. Glucose levels typically rise quickly after stress, consistent with its role as an early stress marker, but may have returned to baseline within approximately two hours, which is consistent with other studies^76^. In contrast, lactate accumulated later and remained elevated at the time of sampling, suggesting an ongoing energetic demand met by anaerobic metabolism, possibly due to the unresolved stressor, such as the injection, or an acute unknown effect of aspirin. To clarify the cause of these effects, future studies should include saline (NaCl) and ethanol (vehicles for aspirin) injection groups, to differentiate between the effects of drug administration, the vehicles used for aspirin (ethanol as a solvent and NaCl as a diluent), and the injection procedure itself^44^. Previous studies have shown that solvents such as ethanol can induce stress or even change the efficacy and action of specific drugs^77^. In addition, some solvents can have negative impacts on behaviour and physiology of decapods^78^. Moreover, the higher lactate levels in the aspirin groups might be either due to the saline solution or the injection itself. There are studies showing that high intoxication with ethanol can increase lactate levels^79^. However, we continued without control animals for the injection due to time constraints and because the final ethanol concentration (0.43 M) was below the concentration (0.5 M) reported to induce mild sedative and anxiolytic-like effects^78^. Although high ethanol doses can cause adverse effects such as limb loss and erratic behaviour^80^, we observed none during injections. Future studies could explore the effects of ethanol alone and the process of the injection of substances.

Immersion in lidocaine may provide a general numbing effect where nociceptors were less responsive to the electric shock since lidocaine exerts its action through blocking sodium channels^40^. Aspirin reduces inflammation by blocking the COX enzymes^46^ and in this case although inflammation from the electric shock might have been reduced, aspirin might potentially cause oxidative stress similarly to the animal or cause various effects in their physiology^81,82^. Overall, lidocaine appears more suitable for alleviating responses to nociceptive stimuli, though its dosage requires careful consideration to avoid sedative or lethal effects^83,84^. These drugs have been rarely studied in decapod crustaceans and so future experimentation should investigate their pharmacological action, duration of effect and other pharmacokinetic parameters as well as testing them on other decapod species since anaesthetic and analgesic drugs can have wildly different species-specific effects (e.g. review of drug use in Rotllant et al.)^57^.

### The effects of handling stress on Norway lobster

Handling is a known stressor for decapod crustaceans^64^. In our study, handling alone induced stress, evidenced by increased activity in all Sham groups immediately after handling, although activity returned to baseline (pre-treatment) values within two hours. Baseline activity of all groups was similar, indicating comparable behaviours before the experimental handling commenced. Immediately after the treatment, all of the Sham groups increased their activity when returned to the home tank, possibly indicating stress. Although activity decreased over time, the effects of handling appeared to subside within one hour, consistent with findings in shore crabs, were minimal handling elicited stress responses^85^. Unlike the activity patterns, grooming did not differ between Control and Sham groups. Increased grooming has only been observed previously in *Palaemon elegans* when a noxious stimulus was introduced^61^. Since handling is stressful but not expected to be painful since the animals were not damaged, this likely explains the lack of grooming-related differences between these treatments. Thus, the behaviour and physiology of the Norway lobster was affected by handling when animals were moved to a test tank and back to their home tank. Our findings indicate that a specific gene (CHH) associated with stress, exhibited altered expression in the central nervous system of Norway lobsters. The expression of CHH was significantly lower in sham group compared to controls, potentially due to a feedback mechanism that suppresses expression following acute stress^86^, however this remains to be tested. Future studies using this species and possibly other decapod crustaceans should perhaps wait for at least one hour before commencing experimentation when moving or handling these animals to ensure they have recovered from the disturbance.

## Conclusions

Electric shock elicited vigorous tail flips in Norway lobsters during the application of the electric shock that were not seen in non-shocked animals. Drugs with analgesic properties reduced this response to the noxious stimulus and could therefore serve as analgesic agents in a laboratory setting. However, the aspirin injection induced side effects, including increased grooming and elevated lactate levels. Lidocaine did not cause any side effects; however, it may have sedative effects at high doses. These findings may support welfare considerations in laboratory research involving stressful or potentially harmful experimental protocols, although further studies are needed to confirm their efficacy, pharmacokinetics, mode of action and safety. By demonstrating both the potential for nociception caused by electric shock and the mitigating effects of analgesics, this study provides a foundation for improving welfare standards for decapods in research, aquaculture, and fisheries.

## Supplementary Information

Below is the link to the electronic supplementary material.

Supplementary Material 1

## Data Availability

The raw data, supplementary materials and some behavioural videos are available at [figshare.com](http:/figshare.com) (accessed on 14 May 2025), https://figshare.com/s/10a65546e4b749904585.
