# Supplementary material for: Effects of analgesia on the response to a noxious stimulus in Norway lobsters (Nephrops norvegicus)
Source: Sci Rep. 2026 Apr 13;16:12190. doi: 10.1038/s41598-026-41687-w (PMC13077011; doi:10.1038/s41598-026-41687-w)
Supplement: Supplementary file 1 — Supplementary Material 1 [file 41598_2026_41687_MOESM1_ESM.docx]

Supplementary Information

Effects of analgesia on the response to a noxious stimulus in Norway lobsters (Nephrops norvegicus)

by

**Eleftherios Kasiouras, Guiomar Rotllant, Albin Gräns, Per Hjelmstedt and Lynne U. Sneddon**

^a^ Department of Biological and Environmental Sciences, University of Gothenburg, PO Box: 463, 405 30, Gothenburg, Sweden; [eleftherios.kasiouras@bioenv.gu.se](mailto:eleftherios.kasiouras@bioenv.gu.se), lynne.sneddon@bioenv.gu.se

^b^ Institut de Ciéncies del Mar (CSIC), Passeig Marítim de la Barceloneta 37, 08003, Barcelona, Spain; guio@icm.csic.es

^c^ Department of Applied Animal Science and Welfare, Swedish University of Agricultural Sciences, PO Box; 463, 405 31, Gothenburg, Sweden; [albin.grans@slu.se](mailto:albin.grans@slu.se), per.hjelmstedt@gmail.com

* Corresponding author.

1. Reliability of the behavioural observations

There were two observers analysing the videos to obtain behavioural data and the inter-rater reliability with the intra class correlation coefficient was 0.975 for the activity data (p< 0.001) and 0.981 for grooming (p< 0.001). These values indicate a strong agreement between the two observers. Additionally, a Pearson correlation was performed for the activity (R= 0.956, p< 0.001) and for grooming (R= 0.971, p< 0.001) showing a strong positive correlation between the two observers. An inter observer reliability test was also performed where six videos were scored again by the same observer. The intra class correlation coefficient test was 0.995, p< 0.001 for activity and 0.997, p< 0.001 for grooming. A Pearsons correlation was also performed and showed a positive correlation for both activity (R= 0.992, p< 0.001) and grooming (R= 0.994, p< 0.001). Thus, the behavioural scoring method was robust and reliable.

2. Behaviour

2.1 Activity

Initially, all the groups were compared together but the full analysis was difficult to interpret because the control was different to most of the other groups and skewed the analysis (Fig. S1). There was an effect of time (F_(3,36)_= 20.303, p< 0.001) and there was an effect of the treatment (F_(6,72)_= 3.206, p= 0.023), but there was no interaction between time and groups (F_(18,216)_= 1.140, p= 0.348). Moreover, the pairwise comparison showed differences between groups that are summarised in Table S1a & b.

**Fig. S1.** Mean (±SE) of the total activity (s) of Norway lobsters, between all groups, across the 4 different time points (n=13, per group) (*p< 0.05, black lines indicate differences between groups, red lines indicate differences across different time points in the same group).

**Table S1.** Pairwise comparison between (**a**) the same treatment (group) across different time points and (**b**) the same time points across different groups.

| (**a**) | |  | |  | |
| --- | --- | --- | --- | --- | --- |
| Treatment | | Time | | p-value | |
| Sham | | after - after 1h | | 0.04 | |
| shocked lidocaine | | after - after 1h | | 0.031 | |
|  | | after- after 2h | | 0.022 | |
| shocked aspirin | | after- after 2h | | 0.019 | |
| (**b**) |  | |  | |  |
| Time | Treatment | | p-value | |  |
| After | control - sham | | 0.035 | |  |
|  | control- shocked+aspirin | | 0.028 | |  |
| After 1h | control- shocked+aspirin | | 0.01 | |  |
| After 2h | control - shocked | | 0.038 | |  |

2.2 Grooming

Similarly for grooming the behavioural data were compared between all the groups across the four different time points. There was an effect of time (F_(3,36)_= 2.978, p= 0.048) and there was no effect of the treatment (F_(6,72)_= 2.360, p= 0.062), nor was an interaction between time and groups (F_(18,216)_= 2.000, p= 0.083). The pairwise comparisons showed differences in the sham + aspirin group between the time points before and after the electric shock (p= 0.014) (Fig. S2).

**Fig. S2.** Mean (±SE) of the total grooming (s) of Norway lobsters, between all 7 groups, across the 3 different time points (n=13, per group) (*p< 0.05, **p< 0.01, ***p< 0.001).

3. Physiological measurements

3.1 Glucose concentration in the haemolymph of lobsters

Glucose concentrations did not differ between the treatments (Control-Sham-Shocked: df= 2, H= 5.532, p= 0.063, Sham-Sham + lidocaine-Sham + aspirin: df= 2, H= 5.547, p= 0.062, Shocked-Shocked + lidocaine-Shocked + aspirin: df= 2, H= 3.670, p= 0.160) when compared with a Kruskal-Wallis test and a Dunn’s test (Fig. S3).

**Fig. S3.** Median (±IQR) of the glucose concentrations (mmol/L), in the haemolymph of the Norway lobster (n=13, per group).

3.2 Differential gene expression in the Suboesophageal ganglia of the Norway lobsters

3.5.1 Brain (Supraoesophageal ganglia)

In the brain the differential expression of the five different genes were compared individually across the seven groups but no significant differences were detected (acetylcholine: df= 6, H= 5.140, p= 0.526; CHH: df= 6, H= 7.683, p= 0.262; GABA B1: df= 6, H= 8.111, p= 0.230; GABA B2: df= 6, H= 5.635, p= 0.465; somatostatin: df= 6, H= 8.187, p= 0.225).

3.5.2 Suboesophageal ganglia

In the suboesophageal ganglia the differential expression of GABA B1 and GABA B2 was different among the groups (GABA B1: df= 6, H= 13.161, p= 0.041; GABA B2: df= 6, H= 12.847, p= 0.046) (Fig. S4a & b). There were no further differences when the groups were compared with Dunn’s test. No more significant differences were detected in any other genes in these ganglia (acetylcholine: df= 6, H= 12.437, p= 0.053; CHH: df= 6, H= 4.469, p= 0.613; somatostatin: df= 6, H= 6.468, p= 0.373) (Fig. S4b).

**Fig. S4.** Box plots showing the differential gene expression of (**a**) GABA B1 and (**b**) GABA B2, from the suboesophageal ganglia (subOG) of Norway lobster across the seven groups. Box plot (within the box, the horizontal lines denote the median values; boxes extend from the 25^th^ to 75^th^ percentiles of each group’s values; the vertical extended lines represent the 95% range of values) (n= 5, per group).
